# A feasibility study on utility of a new digital disposable semirigid nephroscope for Suction mini-percutaneous nephrolithotripsy

**DOI:** 10.1007/s00345-026-06263-x

**Published:** 2026-04-11

**Authors:** Vineet Gauhar, Jaisukh Kalathia, Khi Yung Fong, Steffi Kar Kei Yuen, Bhaskar Somani, Daniele Castellani

**Affiliations:** 1 Department of Urology, Ng Teng Fong Hospital, Singapore, Singapore; 2 Asian Institute of Nephrourology, AINU, Chennai, India; 3 European Association of Urology Section of Endourology (ESEUT), Arnhem, The Netherlands; 4 Fortune Urology Clinic, Botad, Gujarat India; 5 Department of General Surgery, Singapore General Hospital, Singapore, Singapore; 6 S.H. Ho Urology Centre, Department of Surgery, The Chinese University of Hong Kong, Hong Kong, China; 7 Department of Urology, University Hospitals Southampton, NHS Trust, Southampton, UK; 8 Urology Unit, Azienda Ospedaliero-Universitaria Ospedali Riuniti Di Ancona, Via Conca 71, Ancona, 60126 Italy

**Keywords:** Digital, Disposable, Mini PCNL; nephrolithiasis, Single-use semirigid nephroscope, Suction

## Abstract

**Purpose:**

This prospective single center study evaluated the feasibility, perioperative outcomes, and surgeon experience of a novel digital disposable semirigid nephroscope (Redpine^™^) during suction mini-percutaneous nephrolithotomy (SM-PCNL) for renal stones.

**Methods:**

Between March 2024 and March 2025, 328 patients underwent SM-PCNL using the digital disposable nephroscope across institutions. Inclusion criteria encompassed all consenting patients with renal stones managed via prone/supine SM-PCNL (14–22 Fr suction sheath). The digital disposable scope served initially as a cystoscope to place the guidewire and later used for nephroscopy. Data collected included patient/stone characteristics, operative details (lithotripsy time, access technique), complications (Clavien-Dindo), surgeon satisfaction (5-point Likert scale), and stone-free rate (SFR). SFR was assessed via low-dose CT at 30 days (Grade A: no fragments; Grade B: ≤4 mm fragment).

**Results:**

Median stone diameter was 1.7 cm (IQR 1.3–2.3). Median lithotripsy and total operative times were 8 min (IQR 5–16) and 22 min (IQR 20–35), respectively. Intraoperative SFR was 96%. Surgeons reported excellent instrument satisfaction (median Likert score 1/5) for vision, ease of use, and manoeuvrability within the pelvicalyceal system. Complications were rare: major (Clavien ≥ 3) occurred in 0.9% (*n* = 3, including colonic injury, pleural injury, sepsis. One case developed lower pole arteriorvenous fistula, managed by arterial angioembolization. At 30 days, SFR was 100%: Grade A (no fragments) in 47.9% (*n* = 157) and Grade B (≤ 4 mm fragment) in 52.1% (*n* = 171). Reintervention (ESWL) was needed in 1.2% (*n* = 4) within 3 months. Scope tip damage occurred in 0.6% (*n* = 2).

**Conclusion:**

The digital disposable nephroscope demonstrated excellent feasibility and safety in SM-PCNL, facilitating efficient stone clearance time (median 22 min), high SFR, minimal complications, and superior surgeon ergonomics. Its dual cystoscope-nephroscope functionality and plug-and-play design offer significant versatility, supporting its role in advancing minimally invasive PCNL.

## Introduction

Percutaneous nephrolithotomy (PCNL), first introduced in 1976, underwent a substantial advancement from the traditional 30-Fr approach by subsequent innovations in light sources, optics, and imaging technologies. These enabled the development of smaller and more sophisticated scopes, thereby reinforcing the minimally invasive nature of PCNL [1].

Mini-Percutaneous Nephrolithotomy (mini-PCNL or M-PCNL) represents a pivotal progression utilising miniaturised scopes and percutaneous access tracts typically measuring ≤ 22 Fr. The principal aim of mini-PCNL is to reduce morbidity associated with standard PCNL. Evidence demonstrating superior patient recovery outcomes has contributed to the widespread adoption of mini-PCNL as a minimally invasive alternative, broadening its clinical application. This “minimally invasive” trend is increasingly defined not only by incision size or degree of tissue manipulation, but also by patient-centred metrics such as recovery time, pain management, and complication rates [2].

**Fig. 1:**
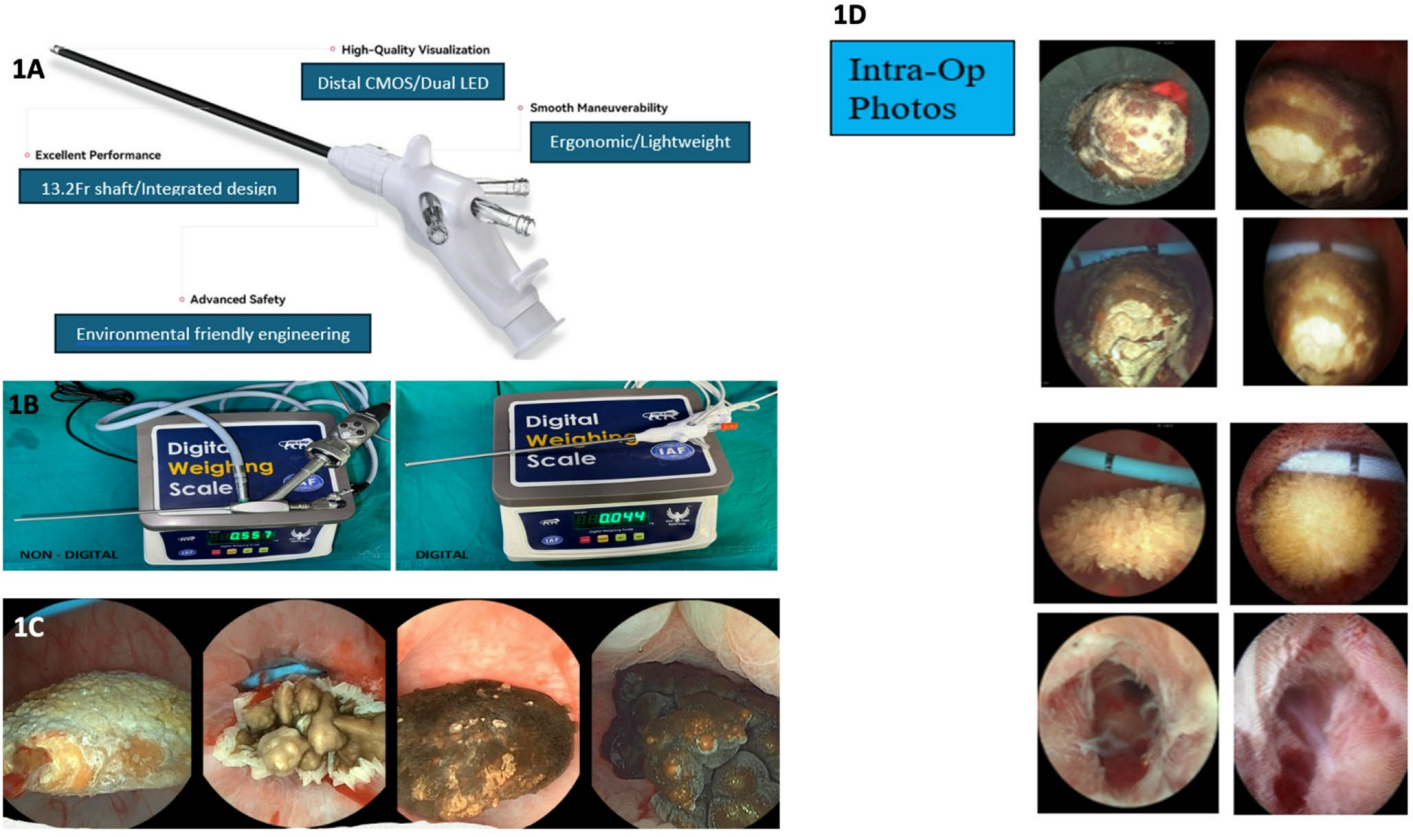
A– Scope design; Fig. 1B - Weight of Conventional Reusable vs. Digital Disposable Nephroscope; Fig. 1C: High-definition image of different stone types; Fig. 1D: High-Definition view of different stone compositions

Digital disposable nephroscopes (DDN) offer key benefits like reduced cross-contamination, stable high-quality images, and easy setup. In 2024, Redpine™ introduced a DDN for M-PCNL, featuring an ergonomic, lightweight, environmentally sustainable pen-grip design. The device includes a chip-on-tip CMOS camera with dual LED illumination, a large working channel in a slim 13.2 Fr shaft and weighs just 44 g with and 34.8 g without cables—significantly lighter than conventional metal models. (44 g vs. 557 g) (Fig. 1A and B), Earlier, in 2008, Gyrus ACMI’s Invisio Smith endoscope, marked a leap as the first digital nephroscope, integrating LEDs and a digital camera to eliminate external light sources while reducing weight [3]. Limited literature suggests this model may not have been commercially successful. Notably, the Redpine™ nephroscope can also function as a cystoscope, aiming to improve PCNL procedures by combining ergonomics, quality, durability and versatility as reported in the first case report on a staghorn stone [4].

Whilst the product is categorized as a disposable product, under strict sterilization practices it is autoclavable and plasma sterilization suitable for multiple uses. This concept of reusable-disposable products is not new in endourology but is the first of its kind on the nephroscope category [5, 6]. The integration of DDN into suction mini-PCNL procedures creates a powerful synergy that significantly enhances stone management. We present our perioperative experience on feasibility and outcomes of using the same in different stone burdens.

## Methods

A prospective, single center, investigator-initiated study enrolled patients between March 2024 and March 2025 by surgeons from Fortune Urology Clinic, Botad, India. Anonymized Data was gathered under IRB-approved registry (IRB#18102023) maintained by the principal site.

### Statistical analysis

Statistical analyses were performed using R Statistical language, version 4.3.0 (R Foundation for Statistical Computing, Vienna, Austria) with *p* < 0.05 indicating statistical significance. Continuous variables were described using median and interquartile range, while categorical variables were described using absolute numbers and percentages.

### Inclusion criteria

Consented patients of all ages with any renal anatomy who underwent prone or supine suction mini PCNL (SM-PCNL) for renal stones were included. SM-PCNL was defined as per International Alliance of Urolithiasis guidelines [7] using a 14-22Fr suction disposable nephrostomy sheath (Amplatz sheath) [8]. Patients who had ureteral stones or underwent non-suction PCNL or had insufficient data obtainable from patient records were excluded. This was strictly in accordance with the Suction Technology Utility in Mini Percutaneous nephrostomy Study (STUMPS) protocol [9].

### Procedure

DDN was initially employed in every patient as a semirigid cystoscope in the supine or lithotomy position. Subsequently the patient was positioned either supine or prone. Guidewires and ureteric catheters for retrograde pyelogram were inserted on the same side under direct visualization with confirmation under fluoroscopy assistance. PCNL access was established using fluoroscopic guidance. If necessary, a second puncture was planned in advance in patients who were pre-operatively thought to need multiple tracts, guidewires were pre-placed in appropriate calyces, and the tract was dilated only if needed. The choice between two types of suction sheaths depended on availability during surgery: either a 16 or 18Fr Clearpetra from Wellead Medical, or a 15Fr, 18Fr, and 21Fr Shah Superperc sheath [10]. At the conclusion of the procedure, the exit strategy was determined according to the surgeon’s preference.

### Baseline and operative characteristics

Patient baseline characteristics, stone features, operative techniques, lithotripsy modality, surgical time, complication, SFR, and reoperation rate were gathered. Stone features on position, site, multiplicity, diameter, volume, and Hounsfield units were assessed using a 2 mm slice unenhanced low-dose CT scan with bone window performed within 6–8 weeks before surgery. Stone diameter of the largest stone and stone volume (ellipsoid formula: length*width*depth*π*0.167) were recorded. Guy’s stone score was recorded. All patients had a preoperative urine culture, and infections were treated appropriately. Anticoagulants/antiplatelets were stopped 3 to 7 days before surgery and restarted as needed.

Two surgeons who independently operated were asked to grade their experience of surgery using the DDN at the end of each case using a 5-point Likert-type scale (1 = excellent; 2 = very good; 3 = good; 4 = average; 5 = poor).

Lithotripsy time was defined as time taken for fragmenting and extracting stone.

Operative time was defined as from placement of ureteral catheter to exit strategy.

The 30-day complications were graded according to Clavien-Dindo score [11]. Pain score was assessed within 24 h of surgery either via teleconsult for day surgery patients or in-person using a 10-point visual analog scale where 1 was the lowest score.

At 4 weeks postoperatively, all patients had a low dose NCCT. Stone-free status was graded as:

- Grade A: no fragment (zero residual fragment, 100% stone free).
- Grade B: single fragment ≤ 4 mm.
- Grade C: single fragment > 4 mm or multiple fragments of any size.

The 30-day secondary perioperative and postoperative outcomes were recorded.

## Results

A total of 328 patients were included. Table 1 summarizes baseline characteristics: median age 46 years (IQR 36–56), 118 (36.0%) males. Most had ASA score 1 (263, 80.2%). Comorbidities: CKD in 53 (16.2%), DM in 4.9%. First-time stone formers made up 276 (84.1%). Nine patients (2.7%) on anticoagulant/antiplatelet therapy were managed as per protocol.

There were 165 (50.3%) left-sided procedures, 162 (49.4%) right-sided procedures, and one simultaneous bilateral SM-PCNL procedure (0.3%). The median largest stone diameter was 1.7 cm (IQR 1.3–2.3), with a median stone volume of 1.1 cm³ (IQR 0.6–3.1). Single stones were observed in 289 patients (88.1%). The median Hounsfield unit for stone density was 1214 (IQR 966–1417). According to the Guy’s stone score (GSS) classification [12], 229 patients (69.8%) were classified as GSS 1, 88 (26.8%) as GSS 2, 10 (3.0%) as GSS 3, and one patient with kyphoscoliosis and partial staghorn in a duplex pelvicalyceal system (0.3%) as GSS 4.

Location wise, mid pole/Interpolar/pelvic stones were most common in 195 patients (59.5%), followed by multiple locations in 82 patients (25.0%), lower pole in 37 patients (11.3%), and upper pole in 14 patients (4.3%). Positive preoperative urine cultures were identified in 188 patients (57.3%), with 321 patients (97.9%) receiving preoperative antibiotics.

Table 2 shows Procedural characteristics. 326 patients (99.7%) underwent SM-PCNL under spinal anesthesia with supine position in 187 procedures (57.0%). Single-tract access was achieved in 307 patients (93.6%), while 21 patients (6.4%) required two tracts. Supracostal access above the 11th rib needed in 54 cases (16.5%). Sequential fascial dilation with two step dilators as preferred tract dilation method in 314 procedures (95.7%), while single-step dilation used in 14 cases (4.3%).

Regarding sheath sizes, 205 procedures (62.5%) utilized sheaths smaller than 16 Fr, 21 procedures (6.4%) employed 16–18 Fr sheaths, and 102 procedures (31.1%) used 21 Fr sheaths. The digital disposable nephroscope demonstrated excellent compatibility with all sheath sizes, with only one case (0.3%) necessitating a sheath change due to patient obesity. Surgeons reported successfully navigating the scope and sheath to visualise all parts of the pelvicalyceal system in 97.56% of cases.

In terms of energy modalities, low-power holmium laser (LPHL) was most frequently utilized in 172 cases (52.9%), followed by Swiss lithoclast in 138 cases (42.5%). High-power holmium laser was employed in 5 cases (1.5%), customised pneumatic lithotripsy device in 9 cases (2.8%), and EMS Lithoclast Trilogy™ in a single bilateral case (0.3%). Stone fragmentation strategies comprised fragmentation and aspiration in 211 cases (64.3%) and combination approaches—including fragmentation, popcorning, and dusting—in 117 cases (35.7%).

Operative efficiency was noteworthy, with a median lithotripsy time of 8 min (IQR 5–16) and a total median operative time of 22 min (IQR 20–35). An intraoperative stone-free rate confirmed by fluoroscopy or visual inspection was attained in 315 cases (96.0%). Use of a flexible nephroscope for calyceal inspection was not required. The digital disposable nephroscope was also successfully utilized as a digital cystoscope in all patients.

Exit strategies, according to surgeon preference, were as follows: tubeless with only stent placement in 231 cases (70.4%), only percutaneous nephrostomy (PCN) in 82 cases (25.0%), both PCN with stent in 6 cases (1.8%), and totally tubeless in 9 cases (2.7%).

Regarding nephroscope durability, scope tip damage associated with lithoclast use occurred in 2 cases (0.6%). Surgeon satisfaction ratings on the Likert scale showed a median score of 1 for all evaluated parameters, including vision, ease of use, and maneuverability within the PCS.

Table 3 presents intraoperative and postoperative outcomes. Intraoperative bleeding was minimal; 326 patients (99.4%) had no bleeding, while 2 patients (0.6%) experienced minor oozing that partially obscured vision but did not interrupt surgery. Postoperative pain scores within 24 h had a median value of 0 (IQR 0–1) on a 10-point visual analog scale.

Complications were uncommon. Major complications (Grade 3–4) included one colonic injury (0.3%), one case with pleural violation with hydro/hemothorax requiring chest tube insertion(0.3%), one case of arterial embolization for a lower pole AVM (0.3%), and one case of sepsis requiring ICU admission (0.3%). Minor complications (Grade 1–2) included fever (0.6%) and persistent haematuria in the first 24 h in 14 cases (4.3%). Blood transfusion was administered in 0.6% of cases, specifically for AVM and sepsis. The median hospital stay was 3 days (IQR 2–4).

At 30-day postoperative CT scan assessment, SFRs were excellent: Grade A (zero residual fragments) in 157 patients (47.9%), Grade B (single residual fragment ≤4 mm) in 171 patients (52.1%) No patients had Grade C outcomes. Reintervention with extracorporeal shock wave lithotripsy (ESWL) was required in only 4 patients (1.2%) within 3 months. Out of the 4 patients, 3 had RF stones at PUJ and one had RF at proximal ureter.

## Discussion

The concept of “minimally invasive surgery” (MIS) in endourology is increasingly defined by patient-centric outcomes (e.g., recovery time, pain, complications) rather than solely by the size of the incision or the degree of tissue manipulation [8]. Whilst this has revolutionized PCNL from a patient outcome perspective, very few technological improvements have earmarked the development in M-PCNL from an operator experience perspective with SM-PCNL [7] and Micro-PERC [11] as last reported significant enhancements to truly create a minimally invasive ergonomic experience via the percutaneous MIS approach.

The introduction of digital flexible ureteroscopes, and by extension, nephroscopes, marked a significant technological leap from their fiber-optic predecessors, which were inherently limited by the fragility of their fiber bundles, often broken during use especially when accessing the lower pole [13].

Digital high-definition image sensors, either Charge-Coupled Devices (CCDs) or Complementary Metal-Oxide-Semiconductors (CMOS), are incorporated directly at the scope’s tip. This “chip-on-tip” design eliminates the need for bulky external camera heads and light sources, making digital scopes lighter and easier to handle, leading to significantly improved ergonomics, thereby reducing surgeon hand fatigue during prolonged procedures [13–16]. Critically, digital scopes deliver superior image quality characterized by higher clarity, resolution, color fidelity, and the absence of the “moiré effect” (a common artifact in fiber-optic imaging), along with enhanced magnification capabilities. This translates to better PCS visualization in flexible ureteroscope studies (90.9% for digital vs. 81.8% for fiber-optic) [14, 15].

To highlight the superior advantage of high-definition image, each time a new DDN was used we intentionally compared this with an available multiple used digital nephroscope in different stone compositions. We compared per operative image quality of stone, instruments inside PCS, PCS itself and exit tract (Fig. 1D). This was however not part of the study protocol. It was clear from images that the new DDN fared several notches higher (Fig. 1C). The high resolution perhaps can make prediction of stone morphology and composition much easier. Whilst this was not assessed as an outcome of this study, there is ongoing research that high-definition image combined with deep learning and artificial intelligence has > 98% positive predictive value for stone recognition [17, 18]. The superior image quality, ease of use when maneuvering in the PCS reflects in the high Likert scale ratings (Table 2).

Surgeons’ skills and experience are critical to SM-PCNL outcomes, from preoperative planning to minimizing complications [19, 20]. Precision equipment miniaturization, suction-aspiration and digital nephroscopes benefits both surgeons and patients in SM-PCNL have improved ergonomics and visualization, although durability data remain limited [1–4, 21]. Safe practice with excellent device performance is why reinterventions (1.2%) and complications (0.9%) were minimal.

Summarizing the benefits:

The new super slim 13.2Fr DDN, weighing just 34.8gm and compatible with any size suction sheath (14–22 Fr), offers unmatched ergonomic and operative versatility. This design eliminates the need for multiple mini PCNL instruments. Serving as a plug-and-play digital cystoscope and nephroscope reduces capital costs, minimizes re-sterilization expenses and additional cystoscopy equipment handling per-operatively requires no extra software or costly monitors. Its single-use design lowers the risk of cross-contamination [22].

Limitations.

This single-centre study evaluates SM-PCNL’s procedural benefits—such as ergonomics, versatility, and image quality. The device stands out for its lightweight, plug-and-play design, compatibility as a digital cystoscope, and elimination of the need for multiple nephroscope sizes. Cost and durability analysis could confirm its potential savings. The lack of a direct comparative arm with reusable digital or fiber-optic nephroscopes precludes definitive conclusions about its relative advantages in image quality or operative efficiency. While the device is currently marketed as disposable, its potential for reuse under strict sterilization protocols introduces unanswered questions regarding performance degradation and economic modeling over multiple procedures [5, 6].

## Conclusion

The high definition Redpine™ disposable digital cysto-nephroscope combined with suction mini PCNL promises an ergonomic, versatile, safe PCNL experience with excellent vision and perioperative efficacy. Further studies can focus on the possible capital cost saving benefit by virtue of its simple plug and play multifunctional design.

**Table 1:** Demographics

Age	Disposable nephroscope (*N* = 328)
	46 [36, 56]
Male gender	118 (36.0)
ASA	
1	263 (80.2)
2	64 (19.5)
3	1 (0.3)
DM	16 (4.9)
CKD	53 (16.2)
Anticoagulant/antiplatelet use	9 (2.7)
First time stone former	276 (84.1)
Positive urine culture	188 (57.3)
Preoperative antibiotics	321 (97.9)
Laterality	
Left	165 (50.3)
Right	162 (49.4)
Bilateral	1 (0.3)
Guy’s stone score	
1	229 (69.8)
2	88 (26.8)
3	10 (3.0)
4	1 (0.3)
Single stone	289(88.1)
Hounsfield units	1214 [966, 1417]
Largest stone diameter, cm	1.7 [1.3, 2.3]
Stone volume, cm^3^	1.1 [0.6, 3.1]
Stone location	
Upper pole	14 (4.3)
Mid pole/Interpolar/pelvic	195 (59.5)
Lower pole	37 (11.3)
Multiple locations	82 (25.0)

ASA – American Society of Anaesthesiologists; DM – diabetes mellitus; CKD – chronic kidney disease.

**Table 2:** Procedural characteristics. (Reported as median [interquartile range] or N (%))

Spinal anaesthesia	Disposable nephroscope (*N* = 328)
	326 (99.7)
Supine positioning	187 (57.0)
Number of tracts	
1	307 (93.6)
2	21 (6.4)
Supracostal access (above 11th rib)	54 (16.5)
Tract dilation method	
Sequential fascial dilatation	314 (95.7)
Single step dilatation	14 (4.3)
Sheath size	
< 16 Fr	205 (62.5)
16–18 Fr	21 (6.4)
21 Fr	102 (31.1)
Energy	
LPHL	172 (52.9)
HPHL	5 (1.5)
Lithoclast with suction	1 (0.3)
Lithoclast	138 (42.5)
Pneumatic	9 (2.8)
Stone fragmentation modality	
Fragmentation only	211 (64.3)
Combination (fragmentation+ popcorning + dusting)	117 (35.7)
Lithotripsy time, min	8 [5, 16]
Total operation time, min	22 [20, 35]
Need for sheath change	1 (0.3)
Sheath able to access all of kidney	320 (97.56)
100% intraoperative SFR on fluoroscopy or visual inspection	315 (96.0)
Exit strategy	
PCN only	82 (25.0)
Tubeless with stent	231 (70.4)
PCN + stent	6 (1.8)
Totally tubeless	9 (2.7)
Damage to scope tip	2 (0.6)
Likert scale ratings	
Vision	1 [1, 2]
Ease of use	1 [1, 1]
Manoeuvrability in PCS	1 [1, 1]

LPHL – lower power holmium laser; HPHL – high power holmium laser; SFR – stone-free rate; PCN – percutaneous nephrostomy; PCS – pelvicalyceal system

**Table 3:** Intra and postoperative outcomes. (Reported as median [interquartile range] or N (%))

Intraoperative bleeding after dilatation	Disposable nephroscope (*N* = 328)
No bleeding	326 (99.4)
Oozing partially obscuring vision despite suction but allowing surgery to continue	2 (0.6)
Blood Transfusion (CD2)	2 (0.6)
Embolization (CD3b)	1 (0.3)
Colon perforation managed conservatively using intravenous fluid and antibiotics (CD2)	1 (0.3)
Pneumothorax managed by intercostal draining under local anaesthesia (CD3a)	1(0.3)
Postoperative pain score (within 24 h)	0 [0, 1]
Sepsis needing ICU (CD4)	1 (0.3)
Postoperative fever (> 38.0 °C) managed with antibiotics in the ward (CD2)	2 (0.6)
Bleeding managed using intravenous fluid without need for blood transfusion (CD 1)	14 (4.3)
Postoperative RF grade on 30-day CT scan	
Grade A: zero RF	157 (47.9)
Grade B: single RF ≤4 mm	171 (52.1)
Grade C: RF>4 mm or multiple of any size	0
Day-case PCNL (discharged within 24 h)	96 (29.3)
Hospital stay, days	3 [2, 4]
Reintervention with ESWL only (within 3 months)	4 (1.2)

CD – Clavien Dindo classification; ICU – intensive care unit; CT – computer tomography; RF – residual fragments; ESWL – extracorporeal shockwave lithotripsy

## Data Availability

No datasets were generated or analysed during the current study.
